# Enhanced Protocatechuic Acid Production From Glucose Using *Pseudomonas putida* 3-Dehydroshikimate Dehydratase Expressed in a Phenylalanine-Overproducing Mutant of *Escherichia coli*

**DOI:** 10.3389/fbioe.2021.695704

**Published:** 2021-06-24

**Authors:** Oliver Englund Örn, Stefano Sacchetto, Ed W. J. van Niel, Rajni Hatti-Kaul

**Affiliations:** ^1^Division of Biotechnology, Department of Chemistry, Center for Chemistry and Chemical Engineering, Lund University, Lund, Sweden; ^2^Division of Applied Microbiology, Department of Chemistry, Center for Chemistry & Chemical Engineering, Lund University, Lund, Sweden

**Keywords:** aromatic building block, protocatechuic acid, shikimate pathway, 3-dehydroshikimate dehydratase, allosteric inhibition, proton motive force

## Abstract

Protocatechuic acid (PCA) is a strong antioxidant and is also a potential platform for polymer building blocks like vanillic acid, vanillin, muconic acid, and adipic acid. This report presents a study on PCA production from glucose via the shikimate pathway precursor 3-dehydroshikimate by heterologous expression of a gene encoding 3-dehydroshikimate dehydratase in *Escherichia coli*. The phenylalanine overproducing *E. coli* strain, engineered to relieve the allosteric inhibition of 3-deoxy-7-phosphoheptulonate synthase by the aromatic amino acids, was shown to give a higher yield of PCA than the unmodified strain under aerobic conditions. Highest PCA yield of 18 mol% per mol glucose and concentration of 4.2 g/L was obtained at a productivity of 0.079 g/L/h during cultivation in fed-batch mode using a feed of glucose and ammonium salt. Acetate was formed as a major side-product indicating a shift to catabolic metabolism as a result of feedback inhibition of the enzymes including 3-dehydroshikimate dehydratase by PCA when reaching a critical concentration. Indirect measurement of proton motive force by flow cytometry revealed no membrane damage of the cells by PCA, which was thus ruled out as a cause for affecting PCA formation.

## Introduction

Decoupling of plastic production from fossil feedstock requires the availability of carbon-neutral polymer building blocks from renewable resources that could be suitable replacements for the currently used materials and can fit into the established value chains (Hatti-Kaul et al., 2020). Only a limited number of biobased building blocks, primarily aliphatic, are currently produced at large scale including lactic acid, succinic acid, 1,4-butanediol, and 1,3-propanediol. Finding economically viable biobased aromatic monomers constitutes an enormous challenge. Aromatic building blocks are essential components of many important plastic categories, the important examples being terephthalic acid (TPA) present in poly(ethylene terephthalate) (PET) and several other polyesters, and styrene used in polystyrene. The aromatic groups increase the durability and the possibility of recycling the polymers (Hatti-Kaul et al., 2020). There are ongoing research efforts to produce biobased aromatic building blocks that could be either drop-ins or substitutes for the existing products (Graglia et al., 2015; Noda and Kondo, 2017; Kucherov et al., 2021).

**FIGURE 1:**
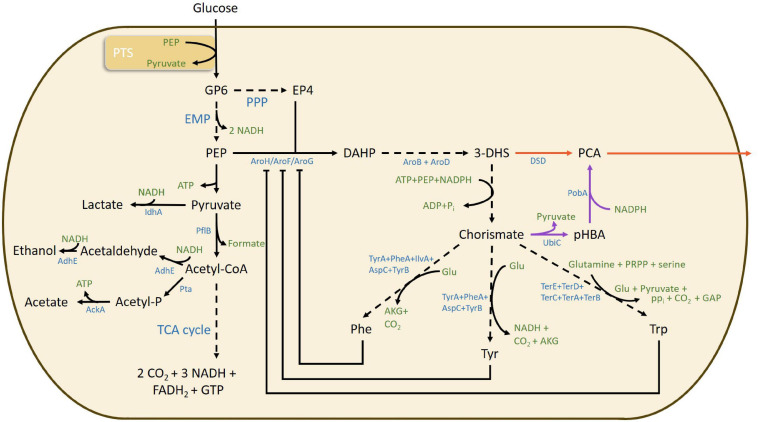
Overview of relevant metabolic pathways, bottlenecks, metabolites, and enzymes involved in protocatechuic acid formation via both DSD (red arrow) and PobA/UbiC (purple arrow) from the shikimate pathway for aromatic amino acid metabolism in *E. coli*. Dashed arrows indicate a multistep enzymatic pathway. 3-dehydroshikimic acid, 3-DHS; acetate kinase, AckA; aldehyde/alcohol dehydrogenase, AdhE; α-ketoglutarate, AKG; 3-dehydroquinate synthase, AroB; 3-dehydroquinate dehydratase, AroD; 3-deoxy-7-phosphoheptulonate synthase, AroF, AroH, and AroG; aspartate aminotransferase, AspC; adenosine triphosphate, ATP; 3-dehydroshikimate dehydratase, DSD; erythrose 4-phosphate, E4P; 3-deoxy-D-arabino-heptulosonic acid 7-phosphate, DAHP; Embden–Meyerhof–Parnas, EMP; flavin adenine dinucleotide reduced form, FADH_2_; glutamate, Glu; guanosine triphosphate, GTP; nicotinamide adenine dinucleotide reduced form, NADH; nicotinamide adenine dinucleotide phosphate reduced form, NADPH; protocatechuic acid, PCA; phosphoenolpyruvate, PEP; pyruvate formate-lyase, pflB; p-hydroxybenzoic acid, pHBA; phenylalanine, Phe; phosphate, P_*i*_; pyrophosphate, PP_*i*_; pentose phosphate pathway, PPP; p-hydroxybenzoate hydroxylase, PobA; 5-phosphoribosyl 1-pyrophosphate, PRPP; phosphate acetyltransferase, Pta; tricarboxylic acid cycle, TCA cycle; tryptophan, Trp; tyrosine, Tyr; tyrosine aminotransferase, TyrB; chorismate lyase, UbiC.

Lignin is the largest natural source of aromatic building blocks, however, separation of the monomers after lignin depolymerization is still a challenge. Several routes to produce biobased TPA have been proposed (Collias et al., 2014). On the other hand, a sugar-based furan building block 2,5-furandicarboxylic acid (FDCA) is being developed as an alternative to TPA (Eerhart et al., 2012; Sousa et al., 2015), and furanics-to-benzene conversion is also being investigated (Kucherov et al., 2021). Several recent publications report on the shikimate pathway used by organisms for production of aromatic amino acids, phenylalanine, tyrosine and tryptophan, as a promising route for obtaining aromatic building blocks for chemicals, and plastics (Rodriguez et al., 2014; Suástegui and Shao, 2016; Lee and Wendisch, 2017; Noda and Kondo, 2017; Averesch and Krömer, 2018; Huccetogullari et al., 2019; Li et al., 2020). Among the various aromatic molecules, protocatechuic acid (PCA; 3,4-dihydroxybenzoic acid) has been identified as a potential platform chemical for several other monomers such as vanillic acid, vanillin, muconic acid, and adipic acid (Pugh et al., 2014). The compound also possesses strong antioxidant and anti-inflammatory properties and has immense pharmacological potential (Kakkar and Bais, 2014). Two recombinant pathways have been described for PCA production (Figure 1). The first pathway uses the enzyme 3-dehydroshikimate dehydratase (DSD) (also abbreviated as 3dhsd, AroZ, AsbF, and QuiC1) to catalyze the dehydration of the intermediate 3-dehydroshikimate (3-DHS) to PCA. The second pathway requires two enzymes - chorismate pyruvate lyase (ubiC) for converting chorismate to *p*-hydroxybenzoate (pHBA) and a NADPH-dependent enzyme *p*-hydroxybenzoate hydroxylase (pobA) for converting pHBA to PCA making it stoichiometrically less favorable (Pugh et al., 2014).

The major metabolic bottleneck for the shikimate pathway has been identified in the synthesis of 3-deoxy-D-arabinoheptulosonate 7-phosphate (DAHP) from phosphoenolpyruvate (PEP) and erythrose-4-phosphate (E4P) coming from glycolysis and pentose phosphate pathways, respectively (Figure 1). The synthesis is constrained in two different ways: (i) the PEP flux to the phosphotransferase system (PTS) (Rodriguez et al., 2014), and (ii) the feedback inhibition of the three DAHP synthase enzymes AroG, AroF and AroH by phenylalanine, tyrosine and tryptophan, respectively (Johansson and Lidén, 2006). Further limitations of PCA production through the shikimate pathway are ascribed to the product toxicity to the cells and inhibition of the DSD enzyme, resulting in termination of the process (Pugh et al., 2014; Shmonova et al., 2020). Addition of 1.5 g/L PCA to *Escherichia coli* culture has been shown to completely impair cell growth (Pugh et al., 2014). The toxicity of PCA could be due to its insertion in the core of the cell membrane, increasing the membrane fluidity and decreasing the structural integrity, as has been observed for other aromatic compounds (Ramos et al., 2002). Studies on DSD of *Corynebacterium glutamicum* have shown the enzyme to be competitively and non-competitively inhibited by PCA (inhibition constants Ki ∼ 0.38 mM and Ki’ ∼ 0.96 mM, respectively) (Shmonova et al., 2020). It is possible to minimize the inhibition by *in situ* removal of the product, which would have an additional benefit of shifting the reaction equilibrium toward product formation (Sayed et al., 2016).

This report presents a study on PCA production through dehydration of 3-dehydroshikimate using recombinant *Pseudomonas putida* DSD in an *E. coli* strain engineered for phenylalanine overproduction by removing the enzymes AroF and AroG, that are inhibited by tyrosine and phenylalanine, respectively, as well as the essential enzymes in the tyrosine and tryptophan biosynthesis pathway (Fox et al., 2008; Peek et al., 2017; Shmonova et al., 2020). The physiological and metabolic effect of PCA production was further evaluated to identify the probable mechanism involved.

## Materials and Methods

### *E. coli* Strains and the Culture Media

*Escherichia coli* strain DH5α was used for construction and long-time storage of assembled plasmids. *E. coli* strains BL21(DE3) and ATCC 31882 (ΔaroF ΔaroG ΔtyrR ΔpheA ΔpheA ΔtyrA ΔtrpE) were used for protein expression and PCA production. LB medium containing per liter 5 g yeast extract, 10 g tryptone, and 5 g NaCl, was used to prepare precultures for batch and fed batch cultures, for growing cells before transformation, DNA and protein isolation and purification. M9 medium containing per liter: 8.5 g Na_2_HPO_4_.2H_2_O, 3 g KH_2_PO_4_, 0.5 g NaCl, 1 g NH_4_Cl, 2 mM MgSO_4_, 0.01 mM CaCl_2_, and 20 g glucose (Neidhardt et al., 1974), was used in initial batch cultivations, and was modified further for use in cultivations for PCA production. The modified M9 medium consisted (per liter) of: 8.5 g Na_2_HPO_4_.2H_2_O, 3 g KH_2_PO_4_, 0.5 g NaCl, 2 g NH_4_Cl, 1 mg thiamine, 2 mM MgSO_4_, 0.1 mM CaCl_2_, 28 μg FeSO_4_, 36 μg (NH_4_)_6_Mo_7_O_24_.4H_2_O, 248 μg H_3_BO_3_, 72 μg CoCl_2_, 24 μg CuSO_4_, 160 μg MnCl_2_, 28 μg ZnSO_4_, and 20 g glucose, unless otherwise specified. In case of nitrogen-limited cultivation, the NH_4_Cl concentration was lowered to 0.2 g/L, while in the phosphate-limited cultivation Na_2_HPO_4_.2H_2_O was omitted and 0.3 g/L KH_2_PO_4_ was used, while maintaining the concentrations of the other components. In certain cultivations, concentrations of all the metal salts were increased four times. In fed-batch experiments, the cultivation was started in a batch mode in the modified M9 medium, and the feed composed of 50 mL of 200 g/L glucose or 50 mL of 200 g/L glucose and 5 mL 200 g/L NH_4_Cl solution added at discrete time points.

### Strain Construction

*Pseudomonas putida* KT2440 was used as a source of the gene coding for DSD. The primers used for gene amplification are described in Supplementary Table 1. The genomic DNA from *P. putida* was extracted using an E.Z.N.A bacterial extraction kit and amplification of the *DSD* gene was done using Phusion High-Fidelity DNA Polymerase (Thermo Fisher Scientific, Waltham, MA, United States) according to the manufacturer’s specifications. The primers also added overhang sequences for two restriction enzymes, *Eco*RI and *Hin*dIII (Thermo Fisher Scientific, Waltham, MA, United States), used for ligation to the plasmid pCDFDuet-1. Ligation was performed using T4 DNA ligase (Thermo Fisher Scientific, Waltham, MA, United States) with 50 ng of linearized vector and five times excess of insert and incubation at 4°C for 16 h, followed by 15°C for 30 min and 25°C for 30 min prior to transformation into competent DH5α cells prepared as described earlier (Hanahan, 1983). Transformation was performed by mixing 50 μL competent cell culture with 2 μL DNA (concentration of 3.5 ng/μL) and incubation on ice for 30 min, after which the cells were subjected to heat-shock for 45 s at 42°C in a water bath and cooled on ice for 2 min before addition of 1 mL LB medium. The cells were incubated for 1 h at 37°C and then 200 μL suspension was plated on LB agar plates supplemented with 50 μg/mL streptomycin. The ligation of *DSD* to the plasmid was confirmed using colony PCR with primers Duet1 Fw/Rev (Supplementary Table 1). Plasmid *pCDFDuet*-*DSD* was transformed into *E. coli* strains BL21(DE3) and ATCC 321882, respectively, using the method described above. Expression of DSD was confirmed using SDS-PAGE. BL21(DE3) and BL21(DE3)-DSD were grown overnight in 50 mL LB medium at 37°C or 30°C until reaching OD_600_ of approximately 5. The cell pellet obtained after centrifugation at 13,000 × *g* at room temperature was re-suspended in TE buffer (100 mM Tris–HCl, 1 mM EDTA, pH 8) and sonicated for 2 min at 24 kHz in a sonicator (Hielscher GmbH, UP 400). The soluble and insoluble fractions were separated by centrifugation at 13,000 × *g*; the insoluble fraction was re-suspended in the same volume of TE buffer, and 10 μL of each sample was loaded on 12% precast acrylamide gel for electrophoresis (Mini-PROTEAN TGX, Biorad).

### Gene Expression and Growth Conditions

*Escherichia coli* DH5α, BL21(DE3), and ATCC 321882 cells were propagated in LB medium, and for growing the cells transformed with pCDFDuet-*DSD* the medium was supplemented with 50 mg/L streptomycin. For production of PCA using BL21(DE3) or ATCC 31882 recombinant strains, M9 or modified M9 medium was used. Batch cultivations in shake flasks and cultivations of the inoculum for batch and fed-batch cultivation were performed at 37°C and 200 rpm, while cultivations in 3 L bioreactors (Applikon Biotechnology, Delft, Netherlands) were maintained at constant temperature of 37°C, pH 7, air flow 1 vvm, and stirring rate of 600 rpm. The pH was controlled by pumping 5 M NaOH solution. In fed-batch cultivations, the conditions were the same except that the stirring rate increased when the dissolved oxygen tension (DOT) value dropped below 40%. Protein expression was induced by addition of 0.1 mM IPTG at the start of the cultivation, unless otherwise specified. Two milliliter culture samples were collected at defined time intervals for monitoring the formation of products and consumption of glucose. In fed-batch experiments, glucose levels were monitored using MQuant glucose test strips (Merek Millipore, MA, United States). *In situ* adsorption of PCA was performed in a normal batch cultivation in the modified M9 medium by suspending 4 g/L of Amberlite 401 IRA (Cl) contained in a Spectra/Por^TM^ 4 (MWCO 12–14 kD) dialysis membrane (Spectrum Chemical Mfg., Corp., NJ, United States).

### Adsorption of PCA

Screening of ion exchange resins Amberlite 400 IRA (Cl), Amberlite 401 IRA (Cl), and Amberlite 904 IRA (Cl) for adsorption and subsequent desorption of PCA was performed using 100 mg resin (pre-swollen in 10 mL water for one-hour) in 10 mL water with PCA (1–10 g/L) and as mixtures with other organic acids (succinic acid and acetic acid at concentrations of 0.1 or 1 g/L each) in 15 mL tubes on a rocking table at room temperature. Subsequently, the resin was washed twice with 10 mL MQ water, and desorption of the adsorbed compounds was tested with 3 × 10 mL eluting solutions (with different concentrations of acetic acid, NaCl and ethanol, respectively) for 30 min each at room temperature. Elution of PCA from Amberlite 401 IRA (Cl) after *in situ* adsorption experiment was performed by treating the resin with 3 × 10 mL of 0.3 M acetic acid solution for 30 min each at room temperature.

### Analytical Methods

#### Cell Dry Weight

The cell dry weight (CDW) of *E. coli* BL21(DE3) was determined by filtering 5 mL cell suspension through a dried and pre-weighed 0.45 μm filter paper (Pall Corp.) in triplicates and then drying overnight at 105°C. The ratio 0.5479 g CDW/OD_600_ and an approximation of the cellular molecular weight of 22.90 g CDW/cmol based on an elemental composition of CH_1_._74_N_0_._24_O_0_._34_S_0_._006_P_0_._005_ (Taymaz-Nikerel et al., 2010) was used in the conversion of measured OD_600_ values to cmol.

#### HPLC Analysis of Substrate and Metabolites

Protocatechuic acid was analyzed using a Dionex HPLC system equipped with a UV/VIS detector and a Phenomenex kinetex 2.6 μm Biphenyl 100 A (50 × 2.1 mm) column for separation using a mobile phase consisting of 93% Solution A containing methanol: acetic acid: water (10:2:88) and 7% Solution B made of methanol: acetic acid: water (90:2:8), at a flow rate of 0.3 mL/min. Glucose, organic acids, and alcohols were analyzed by separation in a Jasco HPLC system equipped with RI detector using a BioRad Aminex HPX87H (Fast Acid) (100 × 7.8 mm) column. The mobile phase was 0.5 mM sulfuric acid used at a flow rate of 0.6 mL/min. The sample injection volume was 10 μL and all samples were filtered through a 0.2 μm filter, diluted 10 times in MQ water to a final volume of 1 mL prior to injecting into the column. The concentration of PCA and other metabolites formed was calculated as gram per liter (g/L) of the medium. Yield of PCA with respect to glucose (Y_*P/S*_) was calculated as mol/mol. Biomass concentration was calculated as dry weight in cmol/L. The yield of PCA with respect to biomass (Y_*P/B*_) was calculated as cmol/cmol. The productivity (g/L/h) was calculated by dividing maximum PCA concentration (g/L) by the time in hours from induction.

#### Flow Cytometry

The effect of PCA on the proton motive force (PMF) of *E. coli* ATCC 31882-DSD was evaluated using a BD Accuri C6 flow cytometer Plus (San Jose, CA, United States) and the dye DiBAC(4)3, as described earlier (Buysschaert et al., 2016). The organism was grown in a bioreactor at pH 7 with or without induction using IPTG. Culture samples were withdrawn in the exponential/early stationary phase (17 h) and late stationary phase (46 h) for measurement of PMF. As positive controls, cells treated with the antibiotic gramicidin D (2, 8, and 20 μg/mL) and heat treatment (30 min at 100°C), respectively, were used. The cell samples were diluted to an OD_600_ of 0.02 and re-suspended in PBS buffer with or without 4 g/L PCA. To stain the cells, 1 μM DiBAC(4)3 was added followed by incubation for 30 min at 37°C. A blue laser of wavelength 480 nm was used for emission and a band filter of 530/30 was used for the excitation measurement. A gate on the forward scatter (FSC) at 20 000 was used to filter out the background noise and 20 000 events were collected for each sample.

## Results

### DSD Gene Expression in *E. coli* BL21(DE3) and PCA Production

**FIGURE 2:**
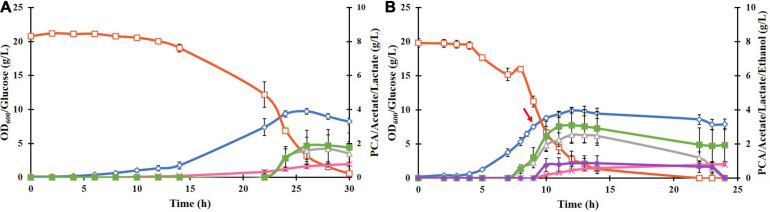
**(A)** Culture parameters of *E. coli* BL21(DE3)-DSD during an aerobic batch cultivation in a bioreactor in modified M9 medium at constant temperature of 37°C, pH 7 and stirring rate of 600 rpm. Expression of DSD gene was done by addition of 0.1 mM IPTG at: **(A)** 0 h, and **(B)** at 9 h after the start of the cultivation shown by a red arrow. The cultivations were performed in duplicates. The symbols denote profiles of cell density measured as OD_600_ (♢), concentrations of glucose (□), PCA (x), acetate (△), lactate (■), and ethanol (◆).

Cloning and transformation of the *P. putida DSD* gene in *E. coli* BL21(DE3) resulted in successful expression of the DSD protein with a His_6_ tag in a soluble form after induction with 0.1 mM IPTG. This was confirmed by SDS-PAGE as a 70 kDa band (Supplementary Figure 1). Cultivation of the recombinant *E. coli* BL21(DE3)-DSD in the normal M9 medium in shake flasks resulted in low cell density (OD_600_ value of 2) and PCA titer (0.2 g/L). But when supplemented with trace metals and thiamine, the OD_600_ and PCA titer increased to 8 and 0.8 g/L, respectively (Figure 2A), and the culture turned black toward the end of the cultivation at 27 h compared to the pale-yellow color of the M9 culture. Such a color change could be attributed to the photochemical oxidation of PCA catalyzed by the metal ions like Fe^3+^ present in the medium (Guo et al., 2020). Yet another likely reason is that the DSD due to its high sequence identity with hydroxyphenylpyruvate dioxygenase (HPPD), catalyzes conversion of 4-hydroxyphenylpyruvate in the tyrosine catabolism pathway to homogentisate (HGA), which undergoes a spontaneous oxidative dimerization, producing or orchronic pigments (Peek et al., 2017). Accumulation of both homogentisate and or orchronic pigments is known to lead to oxidative stress in human cells (Braconi et al., 2015). The color change was not seen in the normal M9 medium.

Cultivating the strain BL21(DE3)-DSD under anaerobic conditions (no air flow, 80 rpm) showed no color change, but led to slowing down of the growth rate and lower biomass formation as expected, and the PCA yield decreasing to 0.007 mol/mol glucose, which corresponds to a PCA concentration of 0.16 g/L, as compared to 0.06 mol/mol under aerobic conditions. Yet another major difference under anaerobic conditions was an increase in lactate formation from 1.6 to 15 g/L due to the fermentative metabolism of glucose (result not shown).

Regardless of the time of inducing the gene expression, whether right at the start of the cultivation or later when the cells were in the late exponential phase, the PCA formation started in the late exponential phase and continued into the stationary phase (Figures 2A,B, respectively). Late induction resulted in 55% increase in the PCA yield, *i.e.*, from 0.046 ± 0.013 to 0.083 ± 0.003 mol/mol glucose (Figure 2B), however, the final PCA titer was lowered from 0.804 ± 0.25 to 0.764 ± 0.117 g/L as less glucose was available during the PCA production phase. The PCA titer was deemed to be a more important variable to maximize compared to the yield, as the use of inducible promoters is impractical in industrial production and that the toxicity/inhibitory effect of PCA as a function of the titer was one of the major factors thought to limit its formation. Hence, in further experiments, cells were induced at the start of cultivations.

### PCA Production Using *E. coli* ATCC 31882-DSD

**FIGURE 3:**
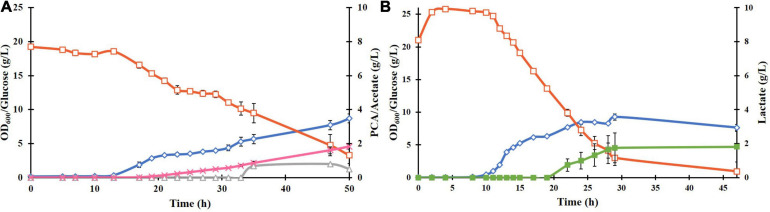
Batch cultivation of *E. coli* ATCC 31882-DSD with the modified M9 medium in a bioreactor at pH 7, 37°C, and constant stirring rate at 600 rpm, with induction of gene expression at 0 h **(A)**. *E. coli* ATCC 31882 was grown as a control **(B)**. The cultivations were performed in duplicates. The symbols represent profiles of cell density measured as OD_600_ (♢), concentrations of glucose (□), PCA (x), acetate (△), and lactate (■).

The plasmid pCSFDuet-*DSD* was then transformed into *E. coli* ATCC 31882, a phenylalanine overproducing strain in which the feedback inhibited enzymes AroF and AroG were removed and so was also the ability to produce tyrosine and tryptophan. Growth under the same conditions as BL21(DE3)-DSD resulted in a higher PCA titer of 1.8 ± 0.28 g/L and yield of 0.13 ± 0.031 mol/mol (Figure 3A). There was also a difference between the strains in the amount of by-products formed; absence of lactate formation in *E. coli* ATCC 31882-DSD was especially notable.

Unlike BL21(DE3)-DSD, the strain ATCC 31882-DSD had what appeared to be two phases of growth (Figure 3A). The initial exponential phase was followed by a relatively long period during which there was no notable increase in biomass but PCA production followed by acetate formation was noticed. Subsequently, a more rapid glucose consumption and biomass increase was observed before cell growth leveled off. A similar pause in exponential phase was observed in the parental strain ATCC 31182 and formation of lactate was initiated (Figure 3B), indicating some kind of metabolic shift in this strain.

The effect of nitrogen and phosphorus limitation on cell growth and PCA production of ATCC 31882-DSD was also studied. The nitrogen-limited batch culture showed a large decrease in biomass formation, glucose consumption and PCA formation, while the strain grown under phosphorus limitation displayed nearly similar growth and PCA yields as under non-limited cultivation (0.124 ± 0.015 mol/mol compared to 0.128 ± 0.031) although at a slower rate and with increased acetate formation (Supplementary Figure 2 and Table 1).

**TABLE 1:** Comparison of results on microbial PCA production obtained in this study with those from the literature.

**Organism and strain**	***E. coli* ATCC 31882**	***E. coli* ATCC 31882**	***E. coli* MG1655**	***S. pombe***	***S. cerevisiae*, BY4741**	***E. coli* BL21(DE3)**	***C. glutamicum* ATCC 21420**	***E. coli* NST74**
Pathway determining intermediate	3-DHS	3-DHS	3-DHS	3-DHS	3-DHS	Chorismate	Chorismate	Chorismate
Overexpressed genes	*DSD*	*DSD*	*DSD*	*DSD*	*TKL1, aroY, CatA, DSD*	*Co-culture Strain A: pobA. Strain B: aroE, aroL, aroA, aroC, ubiC aroGfbr, aroB, aroD, and pobR*	*ubiC, pobA*	*ubiC, pobA*
Source of DSD if applicable	*P. putida*	*P. putida*	*C. glutamicum*	*P. pauciseta*	*K. pneumoniae*			
Genes knocked out	*aroF aroG tyrR pheA tyrA trpE*	*aroF aroG tyrR pheA pheAo tyrA trpE*	*aroE*	-	*aro3 aro4*	*xylA tyrA pheA*	-	*pheA*
Maximum titer (g/L)	4.25	1.82	3.9	0.364	0.300	0.641	1.17	0.454
Yield (mol/mol)	0.182	0.128	0.118	0.0104	0.006	0.110	0.008	0.028
Productivity (g/L/h)	0.079	0.036	0.089	0.008	0.004	0.013	0.010	0.005
Cultivation mode	Fed-batch in bioreactor	Batch in bioreactor	Batch in test tubes	Batch in bioreactor	Batch in shake flask	Batch co-culture	Fed-batch in bioreactor	Batch in bioreactor
References	This study	This study	Shmonova et al. (2020)	Hansen et al. (2009)	Curran et al. (2013)	Guo et al. (2020)	Okai et al. (2016)	Pugh et al. (2014)

*aroA, 3-phosphoshikimate 1-carboxyvinyltransferase; aroB, aroC, chorismate synthase; 3-dehydroquinate synthase; aroD, 3- dehydroquinate dehydratase; aroE, shikimate dehydrogenase; aroL, shikimate kinase Gfbr, feedback-resistant 3-deoxy-7-phosphoheptulonate synthase; aro3, 3-deoxy-7-phosphoheptulonate synthase; aro4, 3-deoxy-7-phosphoheptulonate synthase; aroY, PCA decarboxylase; CatA, catechol 1,2-dioxygenase; TKL1, Transketolase; xylA, Xylose isomerase; ubiC, chorismate pyruvate lyase; pobA, p-hydroxybenzoate hydroxylase; *pobR*, p-hydroxybenzoate hydroxylase transcriptional activator; pheA, fused chorismate mutase/prephenate dehydratase; tyrR, Transcriptional regulatory protein; tyrA, fused chorismate mutase/prephenate dehydrogenase; trpE, anthranilate synthase subunit.*

### Fed Batch Cultivation of *E. coli* ATCC 31882-DSD

**FIGURE 4:**
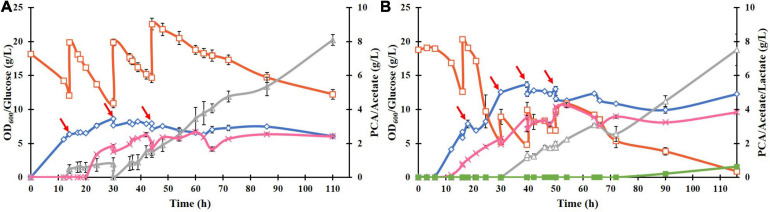
Fed-batch cultivation of *E. coli* ATCC 31882-DSD in 1 liter of the modified M9 medium using a feed of: **(A)** glucose only, and **(B)** glucose and NH_4_Cl. The cultivations were performed in duplicates. Symbols: cell density measured as OD_600_ (♢), concentrations of glucose (□), PCA (x), acetate (△), and lactate (■). The cells were induced for expression of DSD using 0.1 mM IPTG at the start of the cultivation. The feed of approximately 10 g glucose (and 2 g NH_4_Cl) in 50 mL solution was added at discrete time points indicated by red arrows.

To improve the PCA titer, a fed-batch culture of ATCC 31882-DSD was performed using feed with glucose only or with glucose and NH_4_Cl (Figures 4A,B). In the latter case, the PCA reached a maximum titer of 4.25 g/L after 54 h, with a yield of 0.182 mol/mol and a productivity of 0.079 g/L.h. In the absence of nitrogen in the feed, the PCA titer was 2.5 g/L after 110 h, with a final yield of 0.085 mol/mol and productivity of 0.02 g/L/h. Acetate was formed as a major side product in both cases starting at about 30 h of cultivation. In both cases the media turned black at the end of the cultivation.

The biomass formation ceased in both cultures after approximately 40 h, and even the PCA concentration remained almost constant after 54 h or dropped slightly. On the other hand, glucose consumption continued for the duration of the cultivation, resulting in continued formation of acetate reaching the final concentration of 7.5 g/L after 110 h (Figure 4B). The acetate yield in the later phase of the cultivation, *i.e.*, after 54 h in Figure 4B, was 2.0 mol/mol glucose, corresponding to 5.3 g/L. As this is the theoretical maximal yield, it implies complete conversion of glucose to acetate and carbon dioxide during this time, which seems to suggest that the cells switched from running anabolic pathways to catabolic pathways.

### Effect of Trace Metals Addition on Cell Growth and PCA Production by *E. coli* ATCC 31882-DSD

**FIGURE 5:**
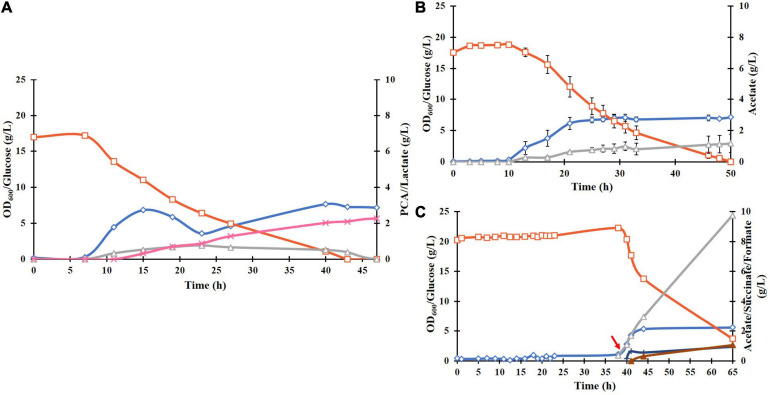
Effect of trace metal concentration and PCA on cell growth and production of metabolites by *E. coli* ATCC 31882-DSD in the modified M9 medium: **(A)** supplemented with four times higher trace metal concentration and induction at 0 h with 0.1 mM IPTG, **(B)** non-induced culture with four times higher trace metal concentration, **(C)** culture as in B and with addition of PCA at 40 h of cultivation. The symbols denote the profiles of cell density measured as OD_600_ (♢), concentrations of glucose (□), PCA (x), acetate (△), succinate (▲), and formate (+).

To investigate if the removal of metal ions due to chelation by PCA was linked to the observed stagnation of biomass and PCA formation and the significant increase in acetate formation (Figure 4B), *E. coli* ATCC 31882-DSD was cultivated in batch mode in the medium at four times higher trace metal concentration but not induced for PCA production. The culture showed an increase in acetate formation and decrease in lactate formation (Figure 5B). Cultivations were then performed by supplementation of 4 g/L PCA to the culture in the mid exponential phase that led to direct increase in acetate formation to 7.9 g/L (Figure 5C), and also formate and succinate to a lower degree. On the other hand, in the induced culture producing PCA in the medium containing higher content of trace metals, acetate formation was lower and PCA titers were similar to that with 1 × trace metals content (Figure 5A).

### Effect of PCA on Proton Motive Force of *E. coli* ATCC 31882-DSD

The possible effect of PCA produced on the PMF of the cells was measured using flow cytometry. The method is based on the use of the fluorescent dye DiBAC(4)3 that enters the cell membrane when the membrane potential changes, which is an indication of a collapsed PMF. Comparison of *E. coli* ATCC 31882 and *E. coli* ATCC 31882-DSD showed that PCA production had no notable effect on the PMF of the cells (Table 2). Also, the addition of 4 g/L PCA to both ATCC 31882 and induced ATCC 31882-DSD cells (after washing) had no effect on the PMF. As a control, gradual addition of the antibiotic gramicidin D led to an increase in the percentage of permeabilized cells. Even the cells subjected to heat treatment did not maintain a PMF, resulting in permeabilization of 99.8% of total cells.

**TABLE 2:** Percentage of cells with collapsed proton motive force as seen by increase of fluorescence by DiBAC(4)3 of *E. coli* ATCC 31882 and *E. coli* ATCC 31882-DSD exposed to 4 g/L PCA, gramicidin and heat, respectively.

**Time**	**Culture**	**Permeabilized cells (%)**	
		**PBS**	**PBS + PCA**
19 h	ATCC 31882	2.6	3.1
19 h	ATCC 31882-DSD	4.6	1.9
43 h	ATCC 31882	7.2	4.7
43 h	ATCC 31882-DSD	7.6	15.0
	Gramicidin (2 μg/mL)	25.7	
	Gramicidin (8 μg/mL)	37.3	
	Gramicidin (20 μg/mL)	43.7	
	Heat treated	99.8	

*Produced PCA at 19 h and 43 h for ATCC 31882-DSD was 0.49 g/L and 5.1 g/L, respectively.*

### Adsorption of PCA to Ion Exchange Resin

Incubation of the PCA containing solution (1–10 g/L) with the ion exchange resins showed complete adsorption to occur within 20 min of incubation. The adsorption capacity of Amberlite 400 IRA (Cl), Amberlite 401 IRA (Cl), and Amberlite 904 IRA (Cl) for PCA was found to be 436, 490, and 312 mg/g, respectively. Elution of the adsorbed PCA from the resins exposed to 10 g/L PCA showed highest degree of elution with 1 M NaCl, 0.3 M acetic acid and 0.5 M acetic acid from Amberlite IRA 400, 401, and 904, respectively. The highest amount of PCA eluted was 386 mg/g_*resin*_ from Amberlite IRA 401 (Cl) by 0.3 M acetic acid, corresponding to an elution yield of 82%.

Subsequently, 4 g/L of Amberlite IRA 401 (Cl) was added to the *E. coli* ATCC 31882-DSD batch culture in the modified M9 medium to study the possibility of *in situ* adsorption of PCA. no PCA adsorption was noted; the PCA concentration in the medium was 1.84 g/L and comparable to cultivation without the resin, and also no PCA could be eluted from the resin (data not shown). No adverse effect on the growth of the bacteria was observed.

## Discussion

Among the various metabolic and regulatory bottlenecks encountered in channeling the carbon flow to aromatic building blocks from the shikimate pathway for aromatic amino acids, this study focused on the effect of relieving allosteric control of DAHP synthesis on PCA production from glucose. Moreover, the route involving direct conversion of DAHP to PCA catalyzed by DSD was preferred over the alternative UbiC/PobA pathway, which is stoichiometrically less favorable and requires one mole each of ATP, NADPH and phosphoenol pyruvate (PEP) for the conversion of 3-DHS to chorismate and one more NADPH further downstream for reduction of *p*-hydroxybenzoic acid (pHBA) to PCA (Figure 1). Also, the use of an additional PEP to form chorismate might be more problematic since it is needed both for the formation of DAHP and for the transport of glucose via the phosphotransferase (PTS) system. Furthermore, UbiC is also allosterically inhibited by pHBA (Siebert et al., 1994). An earlier study making use of the overexpressed PobA/Ubic pathway in *E. coli* showed PCA production with a yield of 0.03 mol/mol glucose only in the strain in which the allosteric regulation was removed (Pugh et al., 2014; Table 1).

Dehydroshikimate dehydratase enzymes play a key role in the degradation of the aromatics quinate and shikimate by soil-associated bacteria (Peek et al., 2017). Multiple DSD variants from fungi, *Acinetobacter* species, *Bacillus* species, and *Pseudomonas* species have been identified. Earlier studies have reported the use of DSD from *Corynebacterium glutamicum*, *Podospora pauciseta*, and *Klebsiella pneumonia* (Hansen et al., 2009; Curran et al., 2013; Shmonova et al., 2020; Table 1). In this study, the *P. putida* DSD gene was cloned and expressed in *E. coli*. The structure of the enzyme has shown the protein to be a fusion of two modules comprising an N-terminal sugar phosphate isomerase like domain that is associated with the DSD activity and a C-terminal hydroxyphenylpyruvate dioxygenase-like domain (Peek et al., 2017). The N-terminal domain of the enzyme possesses limited sequence identity with fungal DSDs and 39.9% identity with the enzyme from *C. glutamicum* (Peek et al., 2017; Shmonova et al., 2020). The DSD from *P. putida* has been reported to have a favorable K_*cat*_/K_*m*_ value of 494.3 × 10^3^ M^–1^ s^–1^ (Peek et al., 2017), compared to the other characterized bacterial DSDs, such as 63.3 × 10^3^ M^–1^ s^–1^ for *C. glutamicum* (Shmonova et al., 2020) and 28.9 × 10^3^ M^–1^ s^–1^ for *Bacillus thuringiensis* (Fox et al., 2008).

Expression of *P. putida* DSD in *E. coli* BL21(DE3) yielded 0.8 g/L PCA when cultivated on glucose, which was comparable to the highest reported (Table 1). We further show that removal of allosteric control of the shikimate pathway resulted in over two-fold increase in PCA titer (1.8 g/L) produced by expression of DSD in *E. coli* ATCC 31882. We investigated the effect of nitrogen and phosphorus limitation on PCA synthesis, as these conditions can influence a shift from oxidative to overflow metabolism (Folsom and Carlson, 2015; Guevara-Martínez et al., 2015). Phosphorus limitation did not affect the PCA production significantly possibly due to the intracellular phosphate pools available. On the other hand, nitrogen limitation was detrimental for PCA production, primarily as a result of reduced biomass formation. Nitrogen is moreover a crucial component needed for production of amino acids and proteins.

Highest PCA titer of 4.25 g/L with a molar yield of 18% from glucose and productivity of 0.079 g/L/h was achieved during fed-batch cultivation of *E. coli* ATCC 31882-DSD using a feed containing glucose and nitrogen in the form of NH_4_Cl. Also, the final cell density was increased from an OD_600_ of 6.1 to 12.3. With the feed containing glucose only, PCA and biomass formation leveled off despite the glucose being continually consumed, and Y_*P*__/__*B*_ remained similar to the cultivation with nitrogen in the feed, *i.e.*, 0.60 cmol/cmol vs. 0.64 cmol/cmol (Supplementary Table 2). A likelihood of the PCA production being inhibited by the removal of Ni^2+^, Mn^2+^, Mg^2+^, and Co^2+^ present in the medium due to chelation by PCA was considered (Yang et al., 2014), as these metal ions are known to increase the activity of *P. putida* DSD (Peek et al., 2017). However, increasing the trace metal concentration four-fold in the medium did not increase the yield of PCA or decrease acetate formation (Figure 5A). On the other hand, the recent report on *C. glutamicum* DSD showed an increased PCA production to 3.9 g/L in the medium supplemented with 10 μM CoCl_2_ (Shmonova et al., 2020).

Once the PCA synthesis ceased in the fed-batch cultivation, the glucose consumption continued and was totally metabolized to acetate. This could be a result of the inhibition of the TCA-cycle or the electron transport chain (ETC) by PCA, resulting in lowered yield of ATP and biomass. Acetate formation, unlike lactate or ethanol, provides an extra ATP, which could be useful as a form of stress response, for example for the efflux of PCA from the cells. Acetate formation was also induced by external supplementation of PCA to the culture. The possibility of PCA interfering in the ETC by inserting itself into the cell membrane and thus increasing the permeability (Pugh et al., 2014), and disrupting the PMF was shown not to be the case as measured by flow cytometry. This suggests that while PCA has an effect on either the TCA or ETC, it is not through metal chelation or lowered PMF.

Hence, while PCA is formed as a metabolite in anabolic metabolism its formation causes a shift to catabolic metabolism in *E. coli*, resulting in the termination of its own production (Figure 4B). The metabolic effect seems to be that acetyl-CoA is preferentially converted through a less energetically favorable route to form acetate rather than through the TCA-cycle although some amounts of the intermediate product succinate were observed. It is clear from Figure 5C that the rise in acetate formation is almost immediate upon addition of PCA, suggesting the mechanism of action is through regulation at the protein level and not through genetic regulation, which would have required a lag period of at least 30 min for the new proteins to be transcribed. PCA inhibition of DSD activity has been reported earlier (Shmonova et al., 2020). However, how PCA mediated enzyme inhibition and effect on glucose metabolism are related is not yet clear and would require further studies to map the metabolism and protein expression to evaluate how the shift to catabolic metabolism is regulated to find means for increasing the tolerance toward the inhibitory product.

While *in situ* product removal is a useful alternative to alleviate the product inhibition, our results showed that while the ion exchanger Amberlite IRA 401 (Cl) was able to adsorb PCA in aqueous solution, it showed no adsorption of the metabolite when included in the culture medium. This could be due to the interference by metal ions and other compounds present in the medium. Nevertheless, our observations are not in agreement with the study that reported a very high PCA titer of 71 g/L when a similar resin AG-1 × 8 was added during production of PCA using *E. coli* modified with DSD (Li et al., 2005). Even without the resin, production of 40 g/L PCA equivalent to 49% mol/mol glucose was reported, which is surprising considering that the growth of *E. coli* is inhibited by 1.5 g/L PCA (Pugh et al., 2014). Our results are more in agreement with the highest yields in *E. coli* reported for aromatic molecules that are not known to be toxic, e.g., 0.27 mol phenylalanine, 0.13 mol tryptophan, 0.43 mol tyrosine, and 0.40 mol salicylate per mol glucose (Zhou et al., 2010; Juminaga et al., 2012; Noda et al., 2016; Chen and Zeng, 2017; Noda and Kondo, 2017).

In conclusion, the study shows that expression of *P. putida* DSD gene in the *E. coli* strain engineered to relieve the allosteric inhibition and grown in a fed-batch mode gave among the highest PCA yield from glucose reported so far. It strongly pinpoints to PCA effectuating product inhibition rather than initiating alterations in the bacterial cell membrane. As a consequence, the inhibition caused a metabolic shift inherent to acetate production. The reduction of acetate formation will most likely be accomplished through applying efficient means for *in situ* removal of PCA to keep it below the critical inhibitory level. Formation of lactate as a by-product, although not significant, can perhaps be avoided by maintaining a high dissolved oxygen tension during the cultivation.

## Data Availability Statement

The original contributions presented in the study are included in the article/supplementary material, further inquiries can be directed to the corresponding author/s.

## Author Contributions

OÖ conceived the project, designed and performed the experiments, and wrote the manuscript. SS designed and performed the experiments. EN directed the project. RH-K conceived and directed the project and wrote the manuscript. All the authors were involved in revising the manuscript.

## Conflict of Interest

The authors declare that the research was conducted in the absence of any commercial or financial relationships that could be construed as a potential conflict of interest.
