# Supplementary material for: Enhanced Protocatechuic Acid Production From Glucose Using Pseudomonas putida 3-Dehydroshikimate Dehydratase Expressed in a Phenylalanine-Overproducing Mutant of Escherichia coli
Source: Front Bioeng Biotechnol. 2021 Jun 24;9:695704. doi: 10.3389/fbioe.2021.695704 (PMC8264583; doi:10.3389/fbioe.2021.695704)
Supplement: Supplementary file 1 [file Presentation_1.pdf]

Supplementary information

Enhanced protocatechuic acid production from glucose using *Pseudomonas putida* 3-dehydroshikimate dehydratase expressed in a phenylalanine-overproducing mutant of *Escherichia coli*

Oliver Englund Örn[1], Stefano Sacchetto[1], Ed W.J. van Niel[2] and Rajni Hatti-Kaul[1]*

[1]Division of Biotechnology, Department of Chemistry, Center for Chemistry & Chemical Engineering, Lund University, Box 124, SE-221 00 Lund, Sweden

[2]Division of Applied Microbiology, Department of Chemistry, Center for Chemistry & Chemical Engineering, Lund University, Box 124, SE-221 00 Lund, Sweden

*** Correspondence:**

Corresponding author

Rajni.Hatti-Kaul@biotek.lu.se

Table S1. Sequences of primers used for amplification of genes from genomic DNA and verification of ligation. Restriction sites are given in bold.

DSD Fw	EcoRI	5'-g**GAATTC**AAGAGATTGAAGCGACGAC-3'
DSD Rev	HindIII	5'-ctc**AAGCTT**AGGCGACTACAGCACCG-3'
Duet1 Fw		5'-GGATCTCGACGCTCTCCCT-3'
Duet1 Rev		5'-GATTATGCGGCCGTGTACAA-3'

Figure S1. SDS-PAGE of the total soluble (s) and insoluble (p) protein of *E. coli* grown at 37 °C or 30 °C. Marking denotes strain and temperature, (1) BL21(DE3) 37 °C, (2) BL21(DE3)-DSD 37 °C, (5) BL21(DE3) 30 °C, and (6) BL21(DE3)-DSD at 30 °C. Protein bands in Precision Plus Protein™ standard of molecular weights 75 kDa and 50 kDa are indicated.

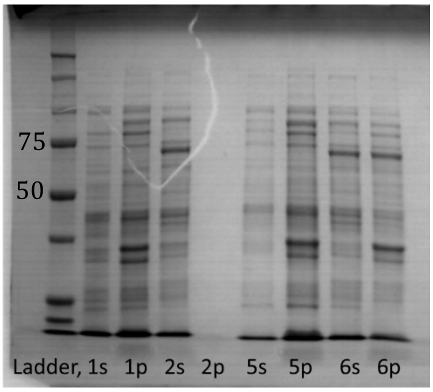

Figure S2. Batch cultivation of *E. coli* ATCC 31882-DSD with phosphorus limitation applied at the start of the cultivation in the Modified M9 medium. In the cultivation a constant temperature of 37 °C, pH 7, and stirring rate of 600 rpm was used. The symbols denote the profiles of cell density measured as OD_{600} (◇), concentrations of glucose (□), PCA (x), and acetate (△).

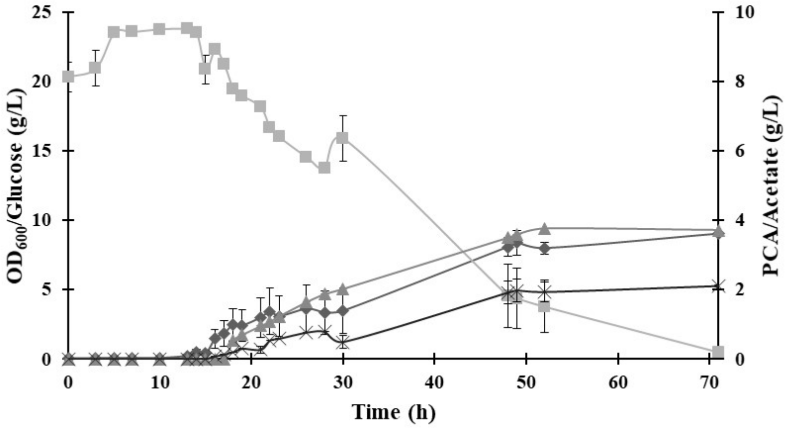

Table S2. Summary of the cultivations preformed in the study.

Strain	Cultivation mode	Maximum titer (g/L)	$Y_{P/S}$ (mol/mol)	$Y_{P/B}$ (cmol/cmol)	Productivity (g/L/h)
***E. coli* BL21(DE3) – DSD**	Batch with 0h induction	0.804 ± 0.250	0.046 ± 0.013	0.181 ± 0.037	0.025 ± 0.008
***E. coli* BL21(DE3) - DSD**	Batch with 9h induction	0.800± 0.090	0.107 ± 0.024	0.478 ± 0.312	0.061 ± 0.013
***E. coli* BL21(DE3) - DSD**	Anaerobic batch	0.164	0.008	0.136	0.002
***E. coli* ATCC 31882-DSD**	Batch with 0h induction	1.823 ± 0.284	0.128 ± 0.031	0.452 ± 0.086	0.036 ± 0.006
***E. coli* ATCC 31882-DSD**	Batch with 19h induction	1.45 ± 0.086	0.117 ± 0.003	0.380 ± 0.046	0.091 ± 0.005
***E. coli* ATCC 31882-DSD**	Batch with nitrogen-limitation	0.173 ± 0.026	0.053 ± 0.013	0.224 ± 0.018	0.005 ± 0.0007
***E. coli* ATCC 31882-DSD**	Batch with phosphorus-limitation	2.100 ± 0.056	0.124 ± 0.015	0.446 ± 0.018	0.030 ± 0.0008
***E. coli* ATCC 31882-DSD**	Fed-batch glucose feed	2.415 ± 0.128	0.092 ± 0.001	0.645 ± 0.042	0.022 ± 0.001
***E. coli* ATCC 31882-DSD**	Fed-batch with glucose and NH_4Cl feed	3.830 ± 0.084	0.120 ± 0.001	0.600 ± 0.038	0.033 ± 0.0007
